# Supplementary material for: Multimodal profiling reveals site-specific adaptation and tissue residency hallmarks of γδ T cells across organs in mice
Source: Nat Immunol. 2024 Jan 4;25(2):343–56. doi: 10.1038/s41590-023-01710-y (PMC10834366; doi:10.1038/s41590-023-01710-y)
Supplement: Supplementary file 1 — Supplementary Note and Supplementary Data 1 [file 41590_2023_1710_MOESM1_ESM.pdf]

nature immunology

Resource https://doi.org/10.1038/s41590-023-01710-y

Multimodal profiling reveals site-specific adaptation and tissue residency hallmarks of γδ T cells across organs in mice

In the format provided by the authors and unedited

Supplementary Note

Two approaches to profile γδ T cells using single-cell multiomics

Sorted γδ cells were subjected to single-cell sequencing using two different approaches. In the first one, γδ T cells from different organs were barcoded with distinct oligonucleotide-conjugated antibodies as well as antibodies identifying various γδ subsets. The transcriptomes and protein expression were simultaneously profiled using single-cell RNA sequencing (scRNA-seq) and TotalSeq™, respectively. In the second approach, simultaneous single-nucleus RNA sequencing (snRNA-seq) and assay for transposase-accessible chromatin using sequencing (scATAC-seq) were performed on pooled γδ T cells from different organs without sample barcoding. Hence, these cells are labelled 'pooled' in **Fig. 1b**.

Implications of cross-organ integration on tissue-specific γδ heterogeneity

In our study, our primary focus was on identifying the common features of tissue adaptation in γδ T cells across various organs. To achieve this, we integrated the datasets by analyzing profiled γδ T cells from different organs using Harmony[1]. Furthermore, for our analysis, we utilized the default resolution provided by the Seurat algorithm[2]. As mentioned in the main text, despite identifying 22 clusters in our scRNA-seq data through unsupervised clustering, we observed that differentially expressed genes in each cluster were not mutually exclusive. Instead, many clusters exhibited differential regulation of identical sets of genes. Consequently, we decided not to increase the clustering resolution. However, it is highly likely that the integration of datasets across organs may have obscured certain specific features of γδ T cells within particular tissues. To investigate this, an analysis of each tissue dataset separately is required to uncover the full spectrum of heterogeneity in γδ T cells within each tissue. We have already observed several examples that reinforce this conclusion. For instance, cluster 15, consisting of proliferating cells, comprises two transcriptionally distinct subsets of cells in the S, G_2 and M phases of the cell cycle (**Fig. 1c, Extended Data Fig. 1g**). Furthermore, we identified a subset of skin *Rorc*$^+$ cells expressing Areg concentrated at a specific coordinate in the UMAP representation within cluster 5 (**Fig. 1c, d, Extended Data Fig. 3h**), and the same holds true for *Rorc*$^+$ cells expressing Vγ4 TotalSeq™ antibody, which exclusively occupy the right corner of clusters 3 and 5 (**Fig. 1c, Extended Data Fig. 2q**). Although conducting such an analysis to unveil the full spectrum of γδ T cell heterogeneity goes beyond the scope of this study due to space limitations, our resource data is freely available for readers to download and explore these questions.

Defining clonotypes for γδ TCR repertoire analysis

We used Cellranger (pipeline Version 7.1.0) to identify distinct clonotypes in the single-cell data. Custom primers were employed to amplify TRG and TRD from single cells. It's important to note that γδ TCR analysis is not a supported workflow in the Cellranger analysis. Therefore, this note provides details on how clonotypes were defined and used in our analysis. Approximately 25,000 cells were loaded into one well of the 10x Genomics chip K for single-cell immune profiling. Experimentally, cells from three female mice (age 8 weeks) were pooled together before sorting. Cells from five different organs (spleen, liver, lung, lymph node, and small intestine) were stained

using hashtag antibodies, pooled, and γδ T cells were FACS sorted for simultaneous single-cell gene expression and TCR profiling. After doublet removal and quality control, 13,753 cells were included in the analysis. Cellranger assigned γδ TCR clonotypes to 4,220 cells, which were then used for TCR repertoire analysis (**Supplementary Table 5**). Among these 4,220 cells, we detected 2,309 unique clonotypes. However, it's important to note that we did not detect fully rearranged productive TRG and TRD chains simultaneously in each cell. This phenomenon is also observed in profiling αβ TCR, primarily because TRA is generally expressed at lower levels than TRB. Therefore, sometimes the TRA chain is not detected. This also appears to be true for the TRD chain, as the median numbers of TRG and TRD UMIs per cell were 11 and 3, respectively. Out of the 2,309 clonotypes, 1,191 comprised both TRG and TRD chains, while 742 and 376 of the clonotypes were annotated based only on TRG and TRD chains, respectively. Although the clonotype annotations provided by Cellranger included a mix, such as those containing both or either of the TRG and TRD chains, they offered better resolution in single-cell TCR data and were therefore used in the analysis. For example, the TRG chain of the most expanded clonotype 1 (CSYGLYSSGFHKVF_CASGYIGGIRATDKLVF) was identical to that of clonotype 49 (CSYGLYSSGFHKVF_NA; NA corresponds to not detected) (**Supplementary Data Fig. 1a, Supplementary Table 5**). However, while clonotype 1 was mainly restricted to *Rorc*$^+$ cells, clonotype 49 was mainly found in *Sell*$^+$ *Ly6c2*$^-$ and *Sell*$^+$ *Ly6c2*$^+$ cells (**Fig. 5c, Supplementary Data Fig. 1a**). The difference between these clonotypes stems from clonotype 1 being paired with a specific TRD chain, while no productive contig of TRD gene could be assigned to clonotype 49. Therefore, clonotype annotation provided by Cellranger in such cases allowed us to have a highly resolved view of cell type-specific clonotypes. Another similar example is clonotype 3 (CASWAGYSSGFHKVF_NA) and clonotype 77 (CASWAGYSSGFHKVF_CALMERESISEGYPTDKLVF), both exhibiting the rearrangement of an identical TRG chain (**Supplementary Data Fig. 1b, Supplementary Table 5**). While both clonotypes are present in the *Gzmb*$^+$ compartment, clonotype 77 was more specific to cluster 2, further indicating the power of using both clonotypes wherever possible (**Fig. 5a, c, Supplementary Data Fig. 1b**). Interestingly, the TRD chain of clonotype 2 (CACWDSSGFHKVF_CGSDIGGSSWDTRQMFF) was identical to that of clonotype 28 (CACWDSSGFHKVF_CGSDIGGSSWDTRQMFF), but it was paired with TRGV6 in clonotype 2 and TRGV5 in clonotype 28 (**Supplementary Data Fig. 1c, Supplementary Table 5**). Importantly, clonotype 2 was mainly restricted to *Rorc*$^+$ cells across organs, while clonotype 28 was mainly present in liver tissue (*Cd160*$^+$ and proliferating cells) (**Fig. 5c, Supplementary Data Fig. 1c**). This further underscores the importance of including either one or both detected clonotypes in the analysis; otherwise, such subtle differences would not be resolved if we only considered either TRG, TRD, or both in the analysis.

Clonotype associated with NKT-like IFN-γ/IL-4 double producers

Previous studies have described a semi-invariant TCR with the TRD nomenclature – TRAV15-1-DV6-1, expressed by NKT-like γδ T cells which produce both IL-4 and IFN-γ, mainly present in the liver and spleen[3, 4, 5, 6]. Although we clearly identified cluster 12 as NKT-like γδ T cell cluster in our dataset, also mainly from the liver and spleen, these cells did not exhibit a productive contig of TRAV15-1-DV6-1 (**Fig. 5a, b, Supplementary Data Fig. 1d, e, Supplementary Table 5**). Cluster 12 comprised 380

cells, out of which we recovered the clonotypes of 174 cells (100 unique clonotypes). In 156 of them, we only detected the TRG chain, indicating that this TRD chain might be lowly expressed in these cells. The cells where both chains were detected, TRDV1 and TRDV2-2, were the predominantly rearranged chains (**Supplementary Table 5**).

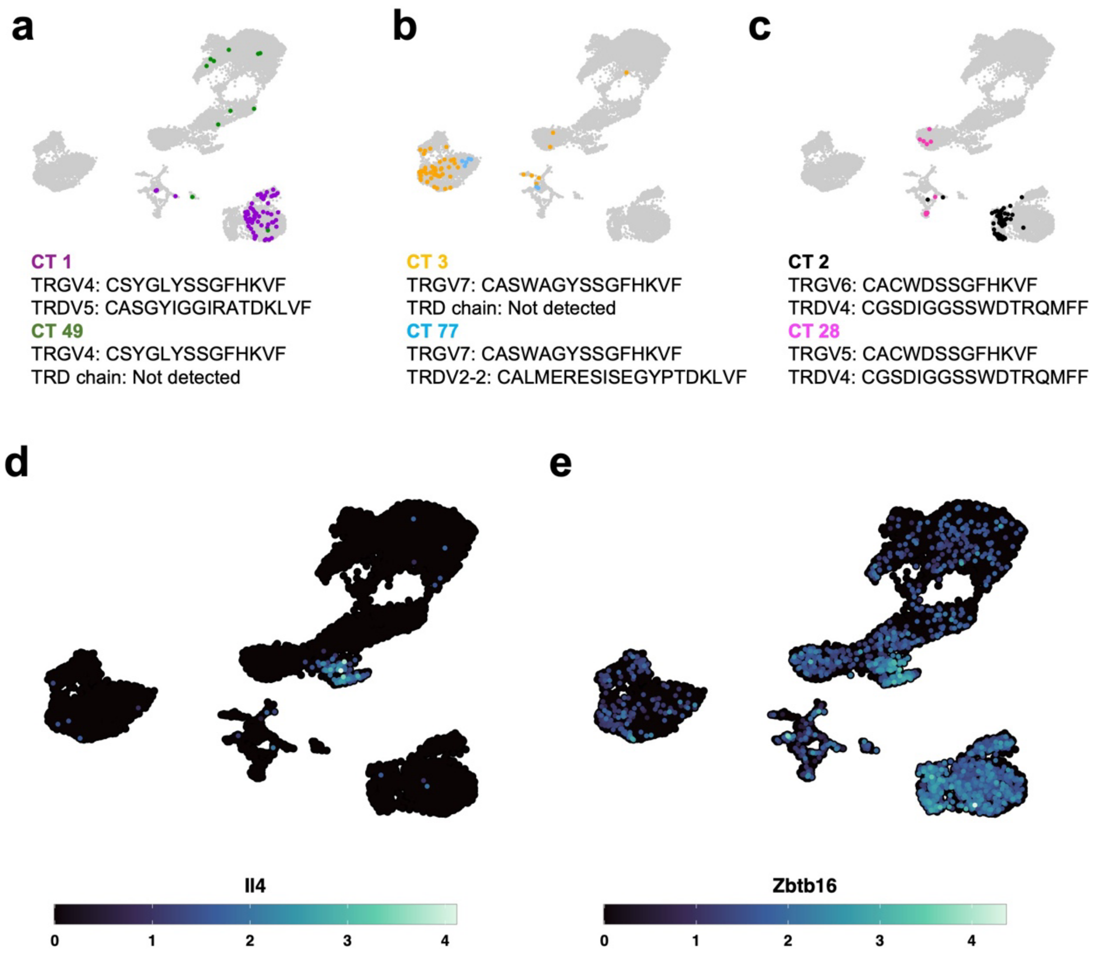

Supplementary Data Figure 1. Defining γδ TCR clonotypes. a-c, UMAP representation highlighting the cells with clonotypes 1 and 49 (**a**), 3 and 77 (**b**) and 2 and 28 (**c**) having identical TRG rearrangement. Each expanded clonotype is represented in a different color. Amino acid sequence of the complementarity-determining region 3 (CDR3) region is further listed. CT, clonotype. **d, e,** UMAP representation showing the normalized transcript counts of *Il4* (**d**) and *Zbtb16* (**e**). Note that the coexpression of both genes is specific to Cluster 12, as shown in **Fig. 5a**.

References

1. Korsunsky, I. *et al.* Fast, sensitive and accurate integration of single-cell data with Harmony. *Nat Methods* **16**, 1289-1296 (2019).

2. Hao, Y. *et al.* Integrated analysis of multimodal single-cell data. *Cell* **184**, 3573-3587 e3529 (2021).

3. Papadopoulou, M., Sanchez Sanchez, G. & Vermijlen, D. Innate and adaptive gammadelta T cells: How, when, and why. *Immunol Rev* **298**, 99-116 (2020).

4. Azuara, V., Levraud, J.P., Lembezat, M.P. & Pereira, P. A novel subset of adult gamma delta thymocytes that secretes a distinct pattern of cytokines and expresses a very restricted T cell receptor repertoire. *Eur J Immunol* **27**, 544-553 (1997).

5. Gerber, D.J. *et al.* IL-4-producing gamma delta T cells that express a very restricted TCR repertoire are preferentially localized in liver and spleen. *J Immunol* **163**, 3076-3082 (1999).

6. Grigoriadou, K., Boucontet, L. & Pereira, P. Most IL-4-producing gamma delta thymocytes of adult mice originate from fetal precursors. *J Immunol* **171**, 2413-2420 (2003).
