# Supplementary material for: Multimodal profiling reveals site-specific adaptation and tissue residency hallmarks of γδ T cells across organs in mice
Source: Nat Immunol. 2024 Jan 4;25(2):343–56. doi: 10.1038/s41590-023-01710-y (PMC10834366; doi:10.1038/s41590-023-01710-y)
Supplement: Supplementary file 2 — Reporting Summary [file 41590_2023_1710_MOESM2_ESM.pdf]

nature portfolio

Corresponding author(s): Sagar
Last updated by author(s): Nov 7, 2023

Reporting Summary

Nature Portfolio wishes to improve the reproducibility of the work that we publish. This form provides structure for consistency and transparency in reporting. For further information on Nature Portfolio policies, see our Editorial Policies and the Editorial Policy Checklist.

Statistics

For all statistical analyses, confirm that the following items are present in the figure legend, table legend, main text, or Methods section.

n/a	Confirmed	
☐	☒	The exact sample size (*n*) for each experimental group/condition, given as a discrete number and unit of measurement
☐	☒	A statement on whether measurements were taken from distinct samples or whether the same sample was measured repeatedly
☐	☒	The statistical test(s) used AND whether they are one- or two-sided<br>*Only common tests should be described solely by name; describe more complex techniques in the Methods section.*
☒	☐	A description of all covariates tested
☐	☒	A description of any assumptions or corrections, such as tests of normality and adjustment for multiple comparisons
☐	☒	A full description of the statistical parameters including central tendency (e.g. means) or other basic estimates (e.g. regression coefficient) AND variation (e.g. standard deviation) or associated estimates of uncertainty (e.g. confidence intervals)
☐	☒	For null hypothesis testing, the test statistic (e.g. F, t, r) with confidence intervals, effect sizes, degrees of freedom and P value noted<br>*Give P values as exact values whenever suitable.*
☒	☐	For Bayesian analysis, information on the choice of priors and Markov chain Monte Carlo settings
☒	☐	For hierarchical and complex designs, identification of the appropriate level for tests and full reporting of outcomes
☒	☐	Estimates of effect sizes (e.g. Cohen's *d*, Pearson's *r*), indicating how they were calculated

Our web collection on statistics for biologists contains articles on many of the points above.

Software and code

Policy information about availability of computer code

Data collection	BD FACSDiva software v6 (Paris) and v8.0.2 (Freiburg) for sorting of cells for single-cell experiments and Cytoflex software (CytExpert) v2.4 for flow cytometry to collect the data for parabiosis FACS validation experiments.
Data analysis	Single-cell data alignment: cellranger-4.0.0, cellranger-6.0.0, cellranger-arc-2.0.1 and cellranger-7.1.0.<br>Single-cell data analysis: R 4.1.3, R 4.2.2, Seurat 4.3.0, Signac 1.8.0, miloR 1.2.0 and scRepertoire 1.7.0<br>FACS data analysis: FlowJo v10.8.0<br>Data representation: Prism v8 and custom codes<br>Codes to reproduce the data analysis and figures are available at: https://github.com/sagar161286/multimodal_gdTcells

For manuscripts utilizing custom algorithms or software that are central to the research but not yet described in published literature, software must be made available to editors and reviewers. We strongly encourage code deposition in a community repository (e.g. GitHub). See the Nature Portfolio guidelines for submitting code & software for further information.

Data

Policy information about availability of data

All manuscripts must include a data availability statement. This statement should provide the following information, where applicable:

- Accession codes, unique identifiers, or web links for publicly available datasets
- A description of any restrictions on data availability
- For clinical datasets or third party data, please ensure that the statement adheres to our policy

The primary read files and the raw counts for all single-cell sequencing datasets reported in this paper are available to download from GEO (accession number: GSE222454, https://www.ncbi.nlm.nih.gov/geo/query/acc.cgi?acc=GSE222454). Processed data can be downloaded from https://github.com/sagar161286/multimodal_gdTcells

Research involving human participants, their data, or biological material

Policy information about studies with human participants or human data. See also policy information about sex, gender (identity/presentation), and sexual orientation and race, ethnicity and racism.

Reporting on sex and gender	n/a
Reporting on race, ethnicity, or other socially relevant groupings	n/a
Population characteristics	n/a
Recruitment	n/a
Ethics oversight	n/a

Note that full information on the approval of the study protocol must also be provided in the manuscript.

Field-specific reporting

Please select the one below that is the best fit for your research. If you are not sure, read the appropriate sections before making your selection.

☒ Life sciences ☐ Behavioural & social sciences ☐ Ecological, evolutionary & environmental sciences

For a reference copy of the document with all sections, see nature.com/documents/nr-reporting-summary-flat.pdf

Life sciences study design

All studies must disclose on these points even when the disclosure is negative.

Sample size	Sample size was mainly determined by accessing the reproducibility of the experiments. To profile gd T cells for single cell experiments, minimum two independent experiments each in Freiburg and Paris were performed. For each independent experiment, 3 mice were used. The data from different independent experiments resulted in the identification of the same clusters/cell types after batch correction reflecting reproducibility. Three independent experiments were performed for single-cell RNA-Sequencing measurements and two independent experiments were performed for simultaneous measurements of RNA and chromatin accessibility at single-cell resolution. Both experiments were performed at two different animal facilities in two different countries (Freiburg, Germany and Paris, Fance). Parabiosis experiments were performed in two independent batches and in each batch 4 pairs of mice (8 in total) were analyzed for tissue resident and exchanging gd T cells. All experiments were highly reproducible.
Data exclusions	While performing single cell analysis, we identified cells of B cell and myeloid cell lineages forming separate and distinct clusters in the dataset. Cells forming these clusters were excluded from the analysis.
Replication	In order to ensure reproducibility, each experiment was performed at least twice independently. After batch correction, we identified all cell types recovered from different single cell experiments. Moreover, we performed such experiments at two different animal facilities to ensure that same cell subsets are present in the mice house in both the facilities. Parabiosis experiments were always performed with 4 mice pairs (8 in total) in each experiment and in single cell experiments, 2-3 mice were always pooled. All replication efforts were successful and the data was highly reproducible.
Randomization	Given age-matched and female mice, mice were randomly assigned to the single-cell and parabiosis experiments.
Blinding	Age-matched and only female mice were used for the experiment. No blinding was applicable as there was no treatment invloved in the experiments.

Reporting for specific materials, systems and methods

We require information from authors about some types of materials, experimental systems and methods used in many studies. Here, indicate whether each material, system or method listed is relevant to your study. If you are not sure if a list item applies to your research, read the appropriate section before selecting a response.

Materials & experimental systems

n/a	Involved in the study
☐	☒ Antibodies
☒	☐ Eukaryotic cell lines
☒	☐ Palaeontology and archaeology
☐	☒ Animals and other organisms
☒	☐ Clinical data
☒	☐ Dual use research of concern
☒	☐ Plants

Methods

n/a	Involved in the study
☒	☐ ChIP-seq
☐	☒ Flow cytometry
☒	☐ MRI-based neuroimaging

Antibodies

Antibodies used

The following antibodies were used at a 1:100 dilution-
Brilliant Violet 421™ anti-mouse TCR β chain Antibody, 109229, BioLegend.
APC Anti-mouse TCR γ/δ Antibody, 118115, BioLegend.
Brilliant Violet 421™ anti-mouse CD62L Antibody, 104435, BioLegend.
PE/Cyanine7 anti-mouse CD160 Antibody, 143009, BioLegend.
Alexa Fluor® 700 anti-mouse CD45.2 Antibody, 109821, BioLegend.
Brilliant Violet 510™ anti-mouse Ly-6C Antibod, 128033, BioLegend.
PerCP-Cy™5.5 Mouse Anti-Mouse CD45.2, 552950, BD.
PE Mouse Anti-Mouse CD45.1, 561872, BD.

The following antibodies were used at a concentration of 1µg per million cells-
TotalSeq™-B 0001 anti-mouse CD4, 100573, BioLegend.
TotalSeq™-B 0002 anti-mouse CD8a, 100783, BioLegend.
TotalSeq™-B 0012 anti-mouse CD117 (c-kit), 105849, BioLegend.
TotalSeq™-B 0073 anti-mouse/human CD44, 103071, BioLegend.
TotalSeq™-B 0093 anti-mouse CD19, 115563, BioLegend.
TotalSeq™-B 0097 anti-mouse CD25, 102067, BioLegend.
TotalSeq™-B 0105 anti-mouse CD115 (CSF-1R), 135543, BioLegend.
TotalSeq™-B 0106 anti-mouse CD11c, 117359, BioLegend.
TotalSeq™-B 0111 anti-mouse CD5, 100645, BioLegend.
TotalSeq™-B 0112 anti-mouse CD62L, 104465, BioLegend.
TotalSeq™-B 0116 anti-mouse Ly-6G/Ly-6C (Gr-1), 108465, BioLegend.
TotalSeq™-B 0118 anti-mouse NK-1.1, 108763, BioLegend.
TotalSeq™-B 0120 anti-mouse TCR β chain, 109261, BioLegend.
TotalSeq™-B 0130 anti-mouse Ly-6A/E (Sca-1), 108149, BioLegend.
TotalSeq™-B 0171 anti-human/mouse/rat CD278 (ICOS), 313551, BioLegend.
TotalSeq™-B 0184 anti-mouse CD335 (NKp46), 137641, BioLegend.
TotalSeq™-B 0191 anti-mouse/rat/human CD27, 124247, BioLegend.
TotalSeq™-B 0197 anti-mouse CD69, 104555, BioLegend.
TotalSeq™-B 0198 anti-mouse CD127 (IL-7Rα), 135055, BioLegend.
TotalSeq™-B 0211 anti-mouse TCR Vγ2, 137715, BioLegend.
TotalSeq™-B 0212 anti-mouse CD24 M1/69, 101847, BioLegend.
TotalSeq™-B 0225 anti-mouse CD196 (CCR6), 129827, BioLegend.
TotalSeq™-B 0227 anti-mouse CD122 (IL-2Rβ), 105913, BioLegend.
TotalSeq™-B 0250 anti-mouse/human KLRG1 (MAFA), 138435, BioLegend.
TotalSeq™-B 0377 anti-mouse CD197 (CCR7), 120133, BioLegend.
TotalSeq™-B 0426 anti-mouse CD192 (CCR2), 150633, BioLegend.

anti–FcR 2.4G2 (Institut Curie, produced in house, 0.25 µg per million cells)

Validation

All validations are performed by the manufacturer from where the antibodies were bought. BioLegend mentions that each lot of the antibody is quality control tested by immunofluorescent staining with flow cytometric analysis. For flow cytometric staining, the suggested use of is ≤ 0.5 µg per million cells in 100 µl volume. BD states that all flow cytometry reagents are titrated on the relevant positive or negative cells. To save time and cell samples for researchers, test size reagents are bottled at an optimal concentration with the best signal-to-noise ratio on relevant models during the product development. To ensure consistent performance from lot-to-lot, each reagent is bottled to match the previous lot MFI.

Animals and other research organisms

Policy information about studies involving animals; ARRIVE guidelines recommended for reporting animal research, and Sex and Gender in Research

Laboratory animals	Experiments were performed using mice from two different animal facilities - Max Planck Institute of Immunobiology and Epigenetics in Freiburg (Germany) and Curie Institute in Paris (France). Parabiosis experiments were performed in Paris. C57BL/6J mice in Freiburg were obtained from in-house breeding and were kept in the animal facility of the Max Planck Institute of Immunobiology and Epigenetics in specific-pathogen-free (SPF) conditions with a 12h light /12h dark cycle, a temperature ranging between 20-23°C and 60% humidity. For the experiments performed in Paris, CD45.1/1 and CD45.1/2 animals were generated in-house by crossing CD45.1/1 B6 animals to CD45.2/2 RORg�t-GFP B6-MAITCAST mice. At the beginning of the experiments, all mice were 6-10 weeks old and were housed in an SPF facility at the Curie Institute with a 12h light /12h dark cycle, a temperature ranging between 22-24°C and 70% humidity.
Wild animals	No wild animals were used.
Reporting on sex	In order to minimize technical variabilities and batch effects, only females were used in all the experiments. Furthermore, females were chosen to avoid the heightened risk of dominance and injury (as we were collecting the skin and this would be a very strong limitation for using males). And consequently, to avoid the sex effect all subsequent mice used in this study were females.
Field-collected samples	This study did not utilize field-collected samples.
Ethics oversight	All animal experiments in Freiburg were performed in accordance with the relevant guidelines and regulations, approved by the review committee of the Max Planck Institute of Immunobiology and Epigenetics and the Regierungspräsidium Freiburg, Germany. For the experiments performed in Paris, All experiments were conducted in an accredited animal facility by the French Veterinarian Department following ethical guidelines approved by the ethics committee of the Institut Curie CEEA-IC (Authorization APAF1S no. 24245–2020021921558370-v1 given by National Authority) in compliance with the international guidelines.

Note that full information on the approval of the study protocol must also be provided in the manuscript.

Flow Cytometry

Plots

Confirm that:

☒ The axis labels state the marker and fluorochrome used (e.g. CD4-FITC).

☒ The axis scales are clearly visible. Include numbers along axes only for bottom left plot of group (a 'group' is an analysis of identical markers).

☒ All plots are contour plots with outliers or pseudocolor plots.

☒ A numerical value for number of cells or percentage (with statistics) is provided.

Methodology

Sample preparation	Cross-tissue single cell preparation<br>All animals were sacrificed using carbon dioxide or cervical dislocation. All organs were collected fresh (i.e., right after sacrifice) in CO2 independent medium (Gibco) and maintained on ice until processing.<br>Spleen and lymph nodes. To isolate cells from the spleen and lymph nodes, tissues were dissected and placed on a 40-µm cell strainer (Falcon, Corning) kept on a 50-ml tube (Falcon, Corning) and were mashed on the cell strainer using the back of the 1-ml syringe plunger. 10 ml phosphate-buffered saline (PBS) containing 0.5% BSA and 2mM EDTA was continuously added while mashing to collect the single-cell suspension in the 50-ml tube. Collected cells were centrifuged at 400 g for 5 min at 4° C. The pellet was resuspended in 10 ml PBS and passed through the 30-µm nylon filter (CellTrics, Sysmex) kept on a 15-ml tube (Falcon, Corning). Cells were again centrifuged at 400 g for 5 min at 4°C. Afterwards the pellet was resuspended in 100 µl of PBS containing 0.5% BSA and 2mM EDTA for subsequent fluorescence-activated cell sorting (FACS) staining. Red blood cell lysis was performed for splenic samples using red blood cell lysis buffer (RBC lysis buffer, 10x, BioLegend) according to manufacturer's protocol.<br>Skin. Skin single cell suspensions were obtained as previously described46. Briefly, Dorsal skin tissue was dissected (flattened, epidermis side up) and incubated at 37 °C for 45 min in 1 mL of 500 CU Dispase (Corning). The tissue was then chopped in RPMI 1640 GlutaMAX media supplemented with 1 mM sodium pyruvate, 1 mM non-essential amino acids, 50 µM β-mercaptoethanol, 20 mM HEPES, 100 U/mL penicillin, 100 mg/mL streptomycin, 0.5 mg/mL DNase I (all products from Sigma-Aldrich), and 0.25 mg/ml Liberase TL (Roche) and incubated for 1h45 min at 37 °C in a 5 % CO2 incubator. After filtering on a 40-µm filter kept on a 50 ml tube, cells were washed twice in PBS, BSA 0.5 %, 2 mM EDTA, the cell suspension was removed of skin debris using the cell debris removal solution (Miltenyi) following manufacturer's instructions.<br>Liver and lung. After perfusion and dissection of the liver and lung, the tissues were finely minced and digested using Collagenase D (0.7 mg/ml in PBS) for 30 min at 37ºC on a shaker in Freiburg while in Paris, the Gentlemacs operating system (Miltenyi) with the m_impTumor_01 program was used as described previously21. After washing the cell pellet twice at 400 x g for 5 min, the pellet was resuspended in 8 mL of 44% Percoll density gradient solution and underlaid with 5 ml 67% Percoll density gradient solution. Centrifugation (without breaks) was performed at 1600 x g for 20 min at room temperature. The cell layer containing mononuclear cells at the interface of the 44% and 67% density gradient centrifugation media was removed, transferred and washed. The resulting pellet was resuspended in 100 µl staining buffer (PBS containing 0.5% BSA

and 2mM EDTA).
Intestinal intraepithelial cells. To isolate intraepithelial lymphocytes from the large and small intestine, tissues were dissected, cleaned to remove feces, cut open and chopped into 2 cm pieces. The pieces were treated with 1 mM Dithioerythritol (DTE) to release intra-epithelial lymphocytes (2X, 20 min each at 37ºC, constant shaking). The supernatant was filtered through 70-µm cell strainers (Falcon, Corning) kept on a 50-ml tube (Falcon, Corning) on ice. Cells were washed one with PBS containing 0.5% BSA and 2mM EDTA and a 44% and 67% density gradient centrifugation was performed as described above. After washing, the resulting pellet was resuspended in 100 µl staining buffer (PBS containing 0.5% BSA and 2mM EDTA).

Antibody staining, flow cytometry, and single-cell sorting. A 100 µl of antibody staining solution was prepared in PBS containing 0.5% BSA and 2mM EDTA and added to the isolated cells resuspended in 100 µl staining buffer as described above. Cells were incubated for 20 min on ice, washed thrice with 2 ml of 0.5% BSA in PBS and resuspended in 3 ml after the last wash for cell sorting. The following antibodies were used (all from BioLegend): TCRgd-APC, TCRb-BV421, CD45.1-PE, CD45.2-AF700, CD45.2-PerCP5.5, CD160-PECy7 and CD62L-BV421. Zombie Aqua and Zombie Green fixable viability kits were used to distinguish dead and living cells. Living TCRgd+ single gd T cells were sorted in BSA-coated tubes containing 50 µl of PBS. Using pulse geometry gates (FSC-W × FSC-H and SSC-W × SSC-H), doublets/multiplets were excluded. After the completion of sorting, the cells were processed through the different 10x Genomics workflows.

Instrument

BD FACSAria™ Fusion and BD CytoFLEX Flow Cytometer

Software

BD FACSDiva software v6 (Paris) and v8.0.2 (Freiburg) for sorting of cells for single-cell experiments and Cytoflex software (CytExpert) v2.4 for flow cytometry to collect the data for parabiosis FACS validation experiments.

Cell population abundance

Purity and the viability of gd T cells were validated by running a purity check on a few microliter of the sorted samples using the same flow cytometer used for sorting.

Gating strategy

Firstly, cells were gated using forward and side scatter to identify events corresponding to lymphocytes, doublets were excluded by gating on single cells using forward scatter height vs. area, living cells were selected by negativity for the viability dye (either Aqua L/D or Zombie Green™ Fixable Viability Kit), afterwards gd T cells were sorted using anti-mouse TCR γ/δ antibody to perform single-cell sequencing experiments. For parabiosis experiments, anti-Mouse CD45.1 and CD45.2 were used to distinguish and sort host-derived and partner-derived gd T cells for single cell sorting and validation experiments.

☒ Tick this box to confirm that a figure exemplifying the gating strategy is provided in the Supplementary Information.
